# The impact of environmental risk and platform trust on satisfaction with health qr code use

**DOI:** 10.3389/fpubh.2022.923974

**Published:** 2022-07-19

**Authors:** Guoliang Shi, Guohua Wang

**Affiliations:** College of Public Administration, Huazhong University of Science and Technology, Wuhan, China

**Keywords:** environment risk, perceived quality, platform trust, health QR code, satisfaction

## Abstract

In response to the economic and social hazards posed by the COVID-19 global pandemic, many countries have adopted various information technologies to rapidly track and control the spread of the epidemic. Health Quick Response (QR) codes are emergency measures implemented by the Chinese government in the epidemic environment to balance epidemic prevention and control with recovery of economic and social development, and facilitate public mobility across regions and access to various public spaces. The use of health codes by the public is a practical necessity, but the satisfaction of their use is influenced by multiple factors such as environment, technology, and organization. In this paper, we collected data through a questionnaire to analyze the basic situation of public satisfaction with the use of health QR codes in China and its influencing factors. The results show that perceived quality and platform trust directly affect the satisfaction of health code usage, while environmental risk and platform trust indirectly affect the satisfaction of health code usage through the mediating effect of perceived quality.

## Introduction

The COVID-19 outbreak has posed unprecedented challenges to countries around the world. Governments have had to take proactive and innovative policy measures to quickly control the spread of the new coronavirus and mitigate the social and economic consequences of the epidemic (1, 2). In order to achieve rapid contact tracking and contain the spread of the virus, governments have adopted a variety of information and communication technologies, such as contact tracking software based on Bluetooth, GPS positioning, QR codes, and other technologies (3). For example, QR codes are used in different ways in different COVID apps, including contact tracing, health reports, vaccine reports (including EU standards for cryptographically checking the QR code vaccine data is genuine), “venue chekin” (so that people could be notified either of exposure at a place they visited in the past, or possibly also for risk level currently). China is a typical case of adopting QR code based technology, and since the first adoption of health code in Hangzhou, it has been rapidly spread nationwide. The health QR code system enhances the government's ability to collect and analyze individuals' past geographic location data and exposure history to classify their risk of infection. As a result, it enables Chinese city governments to quickly identify and isolate potentially infected individuals. At the same time, the health code completes the replacement of paper-based materials and greatly facilitates public mobility across provinces and access to various public places, thus playing a key role in China's economic and social recovery (4). The “most important weapon” in China's “great achievement” of successfully reopening its economy and society without a major backlash was the health code system (5).

Health codes are technological and policy innovations adopted for emergency management, so many studies focus on both the technical and policy dimensions of health code systems. First, as a close-circuiting application, health codes have both advantages and disadvantages (3). From a technical perspective, health codes can efficiently and conveniently collect, store, and share case data, and accurately identify who the close contacts are. However, health codes may at the same time bring harm to the public's personal privacy and negatively affect public data security (4, 6, 7). At the same time, because health codes require certain technical conditions and capabilities to use, there are certain problems for groups such as the elderly to use health codes (8). Second, as a policy tool for innovative use by local governments, the proliferation and improvement of health codes within China has also become a major topic of research. Due to China's unique institutional and cultural environment, local governments are not only influenced by the policies of the central government when responding to new crown epidemics, but also actively learn from the positive practices of other local governments (9, 10). Based on vertical higher-level pressure and horizontal inter-governmental learning, the inter-provincial diffusion of health codes in China has been characterized by explosive diffusion (11, 12).

In general, many countries have adopted digital tracking software to achieve effective outbreak control, so there is a wealth of research on this type of technical software, but less research on a specific software, perhaps due to the unique institutional and cultural environment of each country. Among the existing studies on health codes, many articles focus on policy or technical issues in the government emergency governance dimension, but rarely analyze the public's awareness and use of health codes from their perspective. Although some articles have analyzed the impact of government response on public acceptance from the perspective of urban governance, and concluded that government response to public demands can effectively enhance public acceptance of health codes now and in the future (4). However, few articles really analyze the influence of the public's satisfaction with health code use from individual dimensions such as their living environment, usage, and cognitive ability. Therefore, this paper focuses on analyzing the impact of the public's satisfaction with the use of health codes from three aspects: the external environment of their lives, their trust in the health code use platform, and their technical perception of health code use.

The rest of the paper progresses as follows: first, we present the theoretical basis of the paper and formulate a reasonable research hypothesis; second, we provide a detailed description of the research design of the paper; third, we present the data results in detail; and finally, the conclusions of the study are discussed in a close manner.

## Theoretical basis and research hypothesis

### Perceived quality and health code usage satisfaction

The Theory of Planned Behavior (TPB) and Technology Acceptance Model (TAM) are the two most basic theoretical analysis frameworks in the study of technology adoption, application and evaluation, but the basic premise that “organizations or individuals have the autonomy to adopt and apply technology” is often implied in the analysis of conventional scenarios. In emergency scenarios, technology adoption often has a strong administrative character in order to better improve the effectiveness of emergency governance. Take health code as an example, the government adopts digital emergency services to promote the prevention and control of epidemic and to facilitate the movement of people to promote smooth economic development. In addition, the health code connects data from the departments of industry and information, public security, and transportation, which is also an information system by nature. Therefore, the study of satisfaction with the use of health codes should not be based on the theory of TPB and TAM, but should choose a more suitable system success model (information success). By assessing the information quality, system quality, and service quality of health codes, the public's satisfaction with the use of health codes will be explored.

System success models, especially the D&M system success model proposed by Delone and McLean have been most used in the study of business intelligence systems (13). The initial D&M model delineates six variables from technical, semantic, and utility levels: system quality (emphasizing ease of use, usefulness, reliability, adaptability, etc.) and information quality (emphasizing personalization, completeness, relevance, readability, and security of information), usage and satisfaction, and personal impact and organizational impact (14). After a decade of development, the authors proposed an improved D&M model with two new variables of service quality (including commitment, empathy, responsiveness, etc., which directly affect the support attitude of system users) and willingness to use, integrating personal and organizational impact into net benefits (15). The core of the system success model is the issue of net benefits of information systems, which is basically consistent with the logic of business organizations pursuing economic benefits. However, some scholars believe that net benefits can be affected by other external factors, so the net benefit variables in the D&M model were excluded from several studies such as knowledge management systems, e-commerce websites, mobile banking, and digital multimedia broadcasting, and two variables, satisfaction and intention to use, were used as substitutes (16–19).

Measuring the success of information systems through satisfaction offers the possibility of applying system success models in the public service sector. In the context of mobile health services, the research verified that perceived quality has a positive and significant impact on satisfaction (20). In a recent study of e-government services, it was also confirmed that perceived quality significantly influences public satisfaction (20, 21). Since this paper focuses on the public's perception of the overall quality of health codes, the concept of “perceived quality” is used to distinguish it from information quality, service quality, and system quality. In addition, in other literature, the concepts of “perceived quality” and “service quality” are used to describe information system quality as a whole, but in this paper, “perceived quality” is used instead. In this paper, the term “perceived quality” is used instead of “perceived quality” for better understanding by readers. Therefore, the following hypotheses are proposed in this paper.

H1: The higher the perceived quality of the health code, the higher the public satisfaction with the use of the health code.

### Environmental risk and health code usage satisfaction

The public's use of health codes is in the context of sudden major public health events, and the public's subjective perception of using health codes is inevitably influenced by the external environment. For areas where the epidemic is serious, the public's use of health codes can effectively cooperate with the government to prevent and control the epidemic, thus reducing the loss to society as well as individuals, and thus psychologically and emotionally they will be willing to actively accept and actively use health codes. For areas where the epidemic does not exist or is not serious, the use of health codes is entirely due to individual needs, and if there is no specific need such as cross-province travel, they may not use health codes, thus questioning the necessity of health code use and thus affecting their subjective perception of health codes. Therefore, this paper incorporates external environmental factors into the model based on the system success model to analyze whether the external environmental risks that the public is exposed to affect their satisfaction with health code usage. During an epidemic, due to the uncertainty of the epidemic and the limitation of individual expertise, the public's perception of the risk of the epidemic is often based on new media platforms such as the Internet rather than offline interpersonal interaction and communication. Since the influence of media on individual risk perception is at the social level rather than at the individual level, i.e., the “impersonal influence hypothesis” (22). Therefore, this paper uses individual risk perceptions at the social level to measure public perceptions of external environmental risks.

Environmental risk perception refers to the public's subjective judgment of the objective environment. And the use of public health codes during the epidemic is exactly the risk coping behavior. Therefore, this paper argues that the higher the environmental risk, the higher the satisfaction with the use of health codes. In addition, as a public product and service with “strong contingency,” the public's evaluation of the perceived quality of health codes is inevitably influenced by the risky environment. The higher the environmental risk, the higher the public's evaluation of the perceived quality of health codes. For example, in low environmental risks, the public does not care about the information quality of health codes, which is a psychological effect triggered by the overall environmental safety; while in high environmental risks, the public pays more attention to the information quality of health codes, and the accuracy of information quality directly affects whether the public needs home isolation. Therefore, environmental risk may have a positive effect on the perceived quality of health codes and satisfaction. In summary, the following hypotheses are proposed in this paper.

H2a: The higher the perceived environmental risk, the higher the satisfaction with the use of health codes

H2b: The higher the perceived environmental risk, the higher the perceived quality of the health code

H2c: Mediating role of perceived quality in the effect of perceived environmental risk on intention to continue using

### Platform trust and health code usage satisfaction

From the management dimension, health code technology is beneficial to the government to effectively control the spread of epidemic; however, from the service perspective, the use of health code replaces paper materials and complicated administrative approval, which greatly facilitates the travel needs of the public. Therefore, in an environment where the epidemic is rampant, health codes can be a public service provided by the government in emergency scenarios to serve the daily life of the public. And public trust in organizations has been an important element in public service research, either as an antecedent variable affecting public services or as an outcome variable of public services. In studies of organizational trust and public service satisfaction, much of the literature has emphasized how public trust in government can affect public service satisfaction. However, in the digital environment, the government often does not have the technological advantage and usually needs to cooperate with enterprises, which provide technological support, so organizational trust in digital public services has to take the technology supplier into account. For example, some scholars have divided public trust in e-government websites into two dimensions: “government trust” and “technology trust,” and verified the direct effect of trust on perceived quality and satisfaction, and the indirect effect of trust on satisfaction through perceived quality (23). However, the effect of technology trust was not significant in this study.

Unlike conventional e-government services, the use of health codes is often more closely linked to technical infrastructure such as cell phones, operating systems, WeChat, and Alipay, and the public's trust in the use of health codes is more oriented toward technical trust, i.e., whether the technology can satisfactorily perform the required tasks. Therefore, this paper selects the concept of “platform trust,” which is more technical in nature, as a measure of organizational trust in public health code use. The “platform” referred to in this paper refers to the common platforms used by health codes—WeChat, Alipay, national government service platforms and local government service platforms, including various social media (e.g., WeChat, Alipay), government APPs, small programs, etc. various forms. Therefore, the public's trust in the platforms used for health codes includes both public service platforms provided by enterprises and public service platforms provided by the government. And platform trust not only has a direct impact on satisfaction (24, 25), but also significantly affects the public's perception of system quality (usefulness) (26). Therefore, this paper proposes the following hypothesis.

H3a: The higher the trust in the platform, the higher the satisfaction with the use of health codes

H3b: The higher the trust in the platform, the higher the perceived quality of the health code

H3c: The mediating role of perceived quality in the effect of platform trust on continued willingness to use

In addition to the perceived quality of the information system, the risk of the external environment, and the trust of the organizational platform that affects the satisfaction of health code usage, individual characteristics (gender, age, education level, etc.) may also have an impact (27). Electronic QR codes are not a new thing in China; they are widely used in people's working life, especially for electronic mobile payment using QR codes in daily life. Therefore, the experience of public's use of mobile payment may affect their acceptance of health code use (28). Finally, the use of health codes may be influenced by the public's different experiences during the epidemic. Therefore, in this paper, respondents' gender, age, education level, mobile payment use experience, and whether they had been in a medium-high risk area were included as control variables.

## Study design

### Data collection

In this paper, a questionnaire was used to collect the basic information of the public on the use of health codes. The questionnaire was compiled through Questionnaire Star (https://www.wjx.cn/), and the web link was formed through the questionnaire service provided by this platform, and the questionnaire filling time was set to be no <180 s, and then the questionnaire link was distributed in social media such as WeChat and QQ, and a total of 79 pre-survey questionnaires were collected. After item analysis and validity check of the pre-survey questionnaire data, the official questionnaire was sent through the sample service provided by the questionnaire star platform. The sample service randomly invites eligible members from a sample pool of 2.6 million to fill out the questionnaire, covering all types of occupations, all age groups and sample members of all identities in China, and conducts systematic and manual control of sample quality. From September 26 to September 30, 2021, a total of 299 valid questionnaires were collected, of which 220 were internal samples of Questionnaire Star.

Among the collected sample data, 52.8% were male and 47.2% were female, which is basically consistent with the gender structure of Chinese Internet users (CNNIC, 2021). The age of respondents was mainly concentrated in the youth group aged 19–39; their education level was mainly undergraduate, accounting for 69.6%; the vast majority of them used mobile payment frequently; and a proportion of 20.4% had been in a medium-high risk area. The specific characteristics of the respondents are shown in Table 1.

**Table 1:** Demographic characteristics and individual experiences of the sample.

**Projects**	**Category**	***N*** **(%)**
Gender	Male	158 (52.8)
	Female	141 (47.2)
Age	Below 19 years old	4 (1.3)
	19–39 years old	268 (89.6)
	40–59 years old	26 (8.7)
	Over 60 years old	1 (0.3)
Education level	High school/junior high school and below	16 (5.4)
	College	47 (15.7)
	Undergraduate	208 (69.6)
	Master and above	28 (9.4)
Whether to use mobile payment frequently	Yes	295 (98.7)
	No	4 (1.3)
Have you ever been in a high risk area	Yes	61 (20.4)
	No	238 (79.6)

### Variable measurement

#### Perceived quality (PQ)

The perceived quality of the health code was measured in three dimensions: system quality, information quality, and service quality, with a 5-point Likert scale (from 1 = strongly disagree to 5 = strongly agree) for each question. For the system quality dimension, Chen's scale (29) was used, with five questions: the health code system is clear and easy to understand; the health code system is responsive; the health code system rarely stops working; the health code system functions well; and the health code system provides a good interaction mechanism. Information quality was also measured using Chen's scale, with five questions: the health code system provides correct and accurate information; the health code system provides complete and sufficient information; the health code system provides detailed and sufficient information; the health code system provides the information I need, and the information provided by the health code system helps me solve problems. The service quality was measured with six question items from Teo et al.'s scale (23): the health code system provides reliable service; provides service when it promises; timely service; responds to public requests; is designed with the public's best interest at heart; and is designed to meet public needs. The mean value of the 16 question items of the appeal was calculated to obtain a composite indicator of the perceived quality of the health code.

#### Environmental risk (ER)

The perceived risk of the epidemic at the societal level was measured using a 5-point Likert scale (from 1 = strongly disagree to 5 = strongly agree) by Li et al. (30) on four items: public in other areas are at risk of COVID-19 infection; public in other areas are at risk of COVID-19 infection; COVID-19 may Seriously endanger the health of the public in other areas; COVID-19 may seriously endanger the lives of the public in other areas. By calculating the mean value of the four question items of the appeal, the composite indicator of the perceived quality of health code was obtained.

Platform trust (PT). Drawing on Chen Hong et al.'s classification of “platform trust” (31), and taking into account the actual situation of health code use, it was divided into four categories: WeChat, Alipay, local platform, and national platform, with a total of four items, using five Richter scales (from 1 = very distrustful to 5 = very trustful): how much do you trust the WeChat platform How much do you trust Alipay; how much do you trust the national government service platform; how much do you trust the local government service platform. By calculating the mean value of the four question items in the appeal, the comprehensive index of platform trust is obtained.

#### Usage satisfaction (US)

Using Kim's 4 measures of “satisfaction” with m-Health services (32) on a 5-point Likert scale (from 1 = strongly disagree to 5 = strongly agree): I am satisfied with the use of health codes; I am satisfied with the use of health codes; I like the use of health codes; I am happy with the use of health codes is happy. The mean value of the four question items of the appeal was calculated to obtain a composite indicator of trust in the platform.

### Data analysis

The results of the reliability analysis showed that the total scale reliability (Cronbach's alpha coefficient) was 0.92, and the reliability (Cronbach's alpha coefficient) of each dimension of perceived quality, environmental risk, platform trust, and usage satisfaction were 0.91, 0.67, 0.74, and Therefore, the reliability of the scales used in this questionnaire is acceptable. The HTMT was used to assess the differential validity between variables, which is a new and more reliable method following the Vernell-Lacke criterion and the cross-loading measure (33). The HTMT values were calculated by Smartpls 3.3.3 software and ranged from 0.23 to 0.81, which is less than the threshold value of 0.85, indicating good scale validity. In addition, to determine whether there is a common method bias due to uniform method and uniform data source, this paper was validated by using Harman's one-way test (34). The percentage of variance of the single factor without rotation was found to be 33.12%, which was lower than the 50% threshold, and therefore there was no serious common method bias.

The data were analyzed for correlation, regression, and mediation using SPSS software. Specifically, the hypothesized direct effects were tested using multiple linear regression, and the mediating effects in the hypotheses were tested by PROCESS 3.4.1 using the bootstrapping method (5,000 replications). Multicollinearity diagnosis was performed by calculating the variance inflation factor (VIF) for each variable. The VIF values for each variable were <3, which is below the threshold value of 10. Therefore, there is no serious problem of multicollinearity.

## Results

### Correlation analysis

The means, standard deviations and correlation coefficients of the core variables in this study are shown in Table 2. According to Table 2, the current environmental risk is high, which basically coincides with the continuous local epidemic outbreak in China; the mean values of public perceived quality and usage satisfaction of health code are high, indicating that the domestic public has a high overall evaluation of health code as a digital emergency service; meanwhile, the platform trust score is also high. In terms of correlation, environmental risk was significantly correlated with perceived quality and usage satisfaction; platform trust was significantly correlated with perceived quality and usage satisfaction; and perceived quality was significantly correlated with usage satisfaction. The results of correlation analysis initially verified the research hypothesis of this paper.

**Table 2:** Table of descriptive statistics and correlation analysis for each variable (*N* = 299).

	**Mean**	**Standard deviation**	**1**	**2**	**3**	**4**
ER	3.948	0.586	1			
PQ	4.071	0.539	0.264^**^	1		
PT	4.243	0.594	0.250^**^	0.658^**^	1	
US	3.962	0.646	0.162^**^	0.665^**^	0.529^**^	1

^**^Indicates significant correlation at p < 0.01.

### Regression analysis

Multiple linear regression was used to analyze the variables and the results are presented in Table 3. According to Table 3, it can be obtained that all the control variables in the models have a non-significant effect on perceived quality and usage satisfaction. According to model 2, environmental risk (β = 0.103, *p* < 0.05) and platform trust (β = 0.570, *p* < 0.001) have a significant positive effect on perceived quality, so H2b and H3b hold; according to model 4, perceived quality (β = 0.683, *p* < 0.001), platform trust (β = 0.179, *p* < 0.01) have a significant positive effect on usage satisfaction, while the effect of environmental risk (β = −0.034, *p* > 0.05) is negative and insignificant, so H1 and H3a are valid and H2a is not.

**Table 3:** Results of multiple linear regression analysis.

**Variables**	**PQ**		**US**	
	**Model 1**	**Model 2**	**Model 3**	**Model 4**
Constants	4.336*** (0.390)	1.405 (0.363)	3.973*** (0.470)	0.363 (0.442)
Gender	0.044 (0.064)	−0.021 (0.048)	0.080 (0.077)	0.030 (0.057)
Age	−0.129 (0.098)	−0.101 (0.074)	−0.057 (0.118)	0.042 (0.088)
Education level	0.008 (0.048)	−0.017 (0.036)	−0.008 (0.058)	−0.011 (0.043)
Mobile Payment Experience	−0.182 (0.279)	0.020 (0.209)	0.013 (0.336)	0.189 (0.249)
Medium to high risk experience	0.069 (0.078)	0.062 (0.059)	0.001 (0.094)	−0.054 (0.070)
ER		0.103* (0.041)		−0.034 (0.050)
PT		0.570*** (0.041)		0.179** (0.063)
PQ				0.683*** (0.070)
US				
*R* ^2^	0.014	0.450	0.005	0.461
F	0.829	33.984***	0.302	31.016***
VIF	1.022 ≤ VIF ≤ 1.082	1.022 ≤ VIF ≤ 1.817

(1) Regression coefficients in the table are unstandardized values, standard deviations in parentheses; (2) *Indicates p < 0.05, **indicates p < 0.01, ^***^indicates p < 0.001, two-tailed test.

### Intermediary analysis

The results of the mediation analysis are shown in Table 4: The indirect effects of environmental risk and platform trust on usage satisfaction through perceived quality were 0.18 and 0.37, respectively, with confidence intervals that did not contain 0. Therefore, the hypotheses H2c and H3c were valid. Further, the total effect of environmental risk on usage satisfaction was 0.1785 (*p* < 0.01) and the direct effect was −0.016 (*p* > 0.05). Since the direct effect was not significant while the total effect was significant, perceived quality fully mediated the effect of environmental risk on usage satisfaction. The total effect of platform trust on usage satisfaction was 0.5761 (*p* < 0.001) and the direct effect was 0.1767 (*p* < 0.01). Since both the direct and indirect effects were significant, perceived quality partially mediated the effect of environmental risk on usage satisfaction, and the indirect effect accounted for about 70% of the total effect.

**Table 4:** Mediated effects and 95% confidence intervals estimated by Bootstrap method.

**Paths**	**Indirect effect estimation**	**95% confidence interval**	
	**(Standardization)**	**Lower**	**Upper**
ER → PQ → US	0.18	0.10	0.25
PT → PQ → US	0.37	0.27	0.50

**Figure 1:**
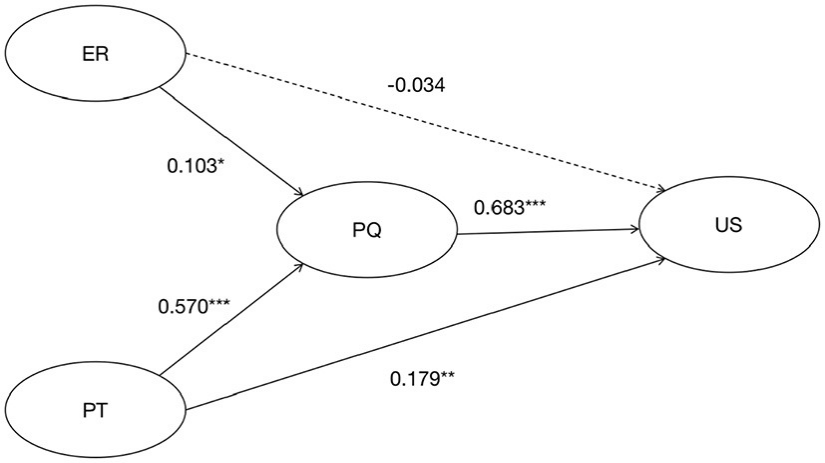
Outcome model of environmental risk, perceived quality, platform trust and usage satisfaction. (1) Dashed line indicates that the relationship is not significant; (2) *indicates *p* < 0.05, **indicates *p* < 0.01, ***indicates *p* < 0.001.

Combining the appeal analysis, we find that all hypotheses are confirmed except for H2a which is not verified. As shown in Figure 1, only the line between the two variables of environmental risk and use satisfaction is dashed, while all other variables are solid lines.

## Conclusion and discussion

Through the empirical survey, it was found that the Chinese public is more satisfied with the use of health codes. This is consistent with the “willingness of the public to use digital contact tracing APPs in the UK, France, Italy, the US and Germany” (35), which indicates that the global public is more supportive of digital outbreak prevention and control, and the public is willing to accept and use such software to actively cooperate with the government to control the spread of the epidemic. Of course, this is closely related to the advantages of “more reliable and time-saving” digital contact tracing (36). In addition, according to this paper, the relationship model of environmental risk, perceived quality, platform trust and public health code use satisfaction can be obtained (see Figure 1). Specifically.

First, perceived quality directly affects public health code usage satisfaction, consistent with the findings of other system success models (29, 37). This is because a high perceived quality health code system implies high perceived benefits, and “perceived benefits directly affect public satisfaction with use” (38). Second, environmental risks do not directly affect public health code usage satisfaction, but have an indirect effect through the mediation of perceived quality. This is generally consistent with the findings of foreign studies on risk perception and digital tracking software (39, 40). The direct effect of environmental risk on satisfaction with health code use is not significant. One possible explanation is that although the multiple outbreaks of local epidemics in China have led to a high public assessment of external environmental risk, this is not the same as the public perceiving themselves as having a high likelihood of being infected by the virus, and a low personal risk state may exist where the public becomes bored with health code use in daily life or sees no need to use health codes, thus affecting the public's satisfaction with health code use. Third, platform trust not only directly and positively affects public satisfaction with health code use, but also indirectly affects satisfaction with continued health code use through the mediation of perceived quality. This is generally consistent with the findings of other research, that studied trust and e-government success (23). It suggests that the public's subjective perceptions of both e-government use in conventional scenarios and health code use in emergency scenarios are influenced by both the quality of the technology and the trustworthiness of the organization behind the technology.

The theoretical contribution of this paper is 2-fold: first, it explores the factors affecting satisfaction with digital emergency technologies from the perspective of the public in a highly uncertain risk environment. Most previous studies on public service satisfaction have focused on e-government services in conventional scenarios, and lack research on digital emergency services in major public health emergencies environments. Second, the system success model is enriched by introducing two variables, external environmental risk and organizational platform trust, into the model to construct a technology-environment-organization framework for analyzing individual public satisfaction with public services. Most previous studies of perceived risk have focused on the uncertainty risk of technology use, and external environmental risk has not been considered. Moreover, since the government is the core subject of digital public services, the measurement of organizational trust is mostly focused on government trust, and the integrated trust analysis of technology and government is rarely considered.

The relevance of this paper is 2-fold: first, the indirect effect of environmental risk on satisfaction with health code use is positive and significant, while there is no significant direct effect. This coincides with the fact that public use of health codes is motivated by the epidemic context, and the perceived risk of the epidemic does not directly affect the public's satisfaction rating of health codes, but rather exerts an indirect influence through perceived quality. The greater the risk of the external environment, the greater the public demand for the quality of health codes, and the greater the importance of the effectiveness of epidemic prevention and control and economic life recovery brought about by the use of health codes. Therefore, the digital emergency services represented by health codes need to pay more attention to the quality of digital technology, i.e., the question of whether they can effectively improve emergency governance. Second, the influence of platform trust on the public's satisfaction with the use of health codes is positive and significant, which indicates that the public has a strong “cognitive and emotional trust” in the convenient services provided by WeChat, Alipay, and government apps (41), thus increasing the satisfaction with the use of health codes on these platforms. In recent years, with the rapid development of Internet information technology, the public's pursuit of quality government services and life services is getting higher and higher, giving rise to more and more convenient digital products and services and public services, such as mobile payment, online shopping, e-government, etc. And while the public enjoys convenient services, it will enhance the level of public trust in enterprises and government, and have an impact on the satisfaction evaluation of services provided by related organizations in the future.

Of course, this study has the following shortcomings: first, this paper is a cross-sectional survey study and lacks a longitudinal epochal analysis. Therefore, there is a lack of time comparison of public satisfaction at various stages of health code development, and it is impossible to understand the interaction between public demand and government supply at different stages, and its impact on public satisfaction with digital emergency services. Second, the research object of this paper is the Chinese public, and lacks cross-country comparisons of countries and regions that have also adopted similar digital emergency services under different systems, environments, and technological conditions. In particular, the providers of apps such as health codes vary considerably across countries, with some being public sector and others being corporate, and thus the effect of trust on attitudes toward use varies considerably across countries. Third, the sample size of this paper is small and mainly consists of young and middle-aged people, and there is a lack of data on the perception and evaluation of digital emergency services for the elderly and children who are “at higher risk of infection” but lack digital skills, so the scope of extrapolation of the findings is limited (42). Follow-up studies should be conducted based on these shortcomings.

## Data availability statement

The original contributions presented in the study are included in the article/supplementary material, further inquiries can be directed to the corresponding authors.

## Ethics statement

Ethics review and approval/written informed consent was not required as per local legislation and institutional requirements.

## Author contributions

GS: research design, methodology, results analysis, and original draft writing. GW: data screening and reviewing and editing. Both authors contributed to the article and approved the submitted version.

## Conflict of interest

The authors declare that the research was conducted in the absence of any commercial or financial relationships that could be construed as a potential conflict of interest.

## Publisher's note

All claims expressed in this article are solely those of the authors and do not necessarily represent those of their affiliated organizations, or those of the publisher, the editors and the reviewers. Any product that may be evaluated in this article, or claim that may be made by its manufacturer, is not guaranteed or endorsed by the publisher.
